# Evolution in Laparoscopic Gastrectomy From a Randomized Controlled Trial Through National Clinical Practice

**DOI:** 10.1097/SLA.0000000000006162

**Published:** 2023-11-23

**Authors:** Sheraz R. Markar, Maurits R. Visser, Arjen van der Veen, Misha D.P. Luyer, Grard Nieuwenhuijzen, Jan H.M.B. Stoot, Juul J.W. Tegels, Bas P.L. Wijnhoven, Sjoerd M. Lagarde, Wobbe O. de Steur, Henk H. Hartgrink, Ewout A. Kouwenhoven, Eelco B. Wassenaar, Werner A. Draaisma, Suzanne S. Gisbertz, Mark I. van Berge Henehouwen, Donald L. van der Peet, Jelle P. Ruurda, Richard van Hillegersberg

**Affiliations:** * Nuffield Department of Surgery, University of Oxford, UK; † Department of Upper Gastrointestinal Surgery, University Medical Center Utrecht, The Netherlands; ‡ Scientific Bureau, Dutch Institute for Clinical Auditing, Leiden, The Netherlands; § Department of Surgery, Catharina Hospital, Eindhoven, The Netherlands; ∥ Department of Surgery, Zuyderland Medical Center, Heerlen and Sittard-Geleen, The Netherlands; ¶ Department of Surgery, Erasmus University Medical Center, Rotterdam, The Netherlands; # Department of Surgery, Leiden University Medical Center, Leiden, The Netherlands; ** Department of Surgery, ZGT Hospitals, Almelo, The Netherlands; †† Department of Surgery, Gelre Hospitals, Apeldoorn, The Netherlands; ‡‡ Department of Surgery, Meander Medical Center, Amersfoort, The Netherlands; §§ Department of Surgery, Amsterdam UMC location University of Amsterdam, Amsterdam, The Netherlands; ∥∥ Cancer Treatment and Quality of Life, Cancer Center Amsterdam, Amsterdam, The Netherlands; ¶¶ Department of Surgery, Amsterdam UMC location Vrije Universiteit, Amsterdam, The Netherlands

**Keywords:** gastric cancer, gastrectomy, laparoscopy

## Abstract

**Objective:**

To examine the influence of the LOGICA RCT (randomized controlled trial) upon the practice and outcomes of laparoscopic gastrectomy within the Netherlands.

**Background:**

Following RCTs the dissemination of complex interventions has been poorly studied. The LOGICA RCT included 10 Dutch centers and compared laparoscopic to open gastrectomy.

**Methods:**

Data were obtained from the Dutch Upper Gastrointestinal Cancer Audit (DUCA) on all gastrectomies performed in the Netherlands (2012–2021), and the LOGICA RCT from 2015 to 2018. Multilevel multivariable logistic regression analyses were performed to assess the effect of laparoscopic versus open gastrectomy upon clinical outcomes before, during, and after the LOGICA RCT.

**Results:**

Two hundred eleven patients from the LOGICA RCT (105 open vs 106 laparoscopic) and 4131 patients from the DUCA data set (1884 open vs 2247 laparoscopic) were included. In 2012, laparoscopic gastrectomy was performed in 6% of patients, increasing to 82% in 2021. No significant effect of laparoscopic gastrectomy on postoperative clinical outcomes was observed within the LOGICA RCT. Nationally within DUCA, a shift toward a beneficial effect of laparoscopic gastrectomy upon complications was observed, reaching a significant reduction in overall [adjusted odds ratio (aOR):0.62; 95% CI: 0.46–0.82], severe (aOR: 0.64; 95% CI: 0.46–0.90) and cardiac complications (aOR: 0.51; 95% CI: 0.30–0.89) after the LOGICA trial.

**Conclusions:**

The wider benefits of the LOGICA trial included the safe dissemination of laparoscopic gastrectomy across the Netherlands. The robust surgical quality assurance program in the design of the LOGICA RCT was crucial to facilitate the national dissemination of the technique following the trial and reducing potential patient harm during surgeons learning curve.

Randomized controlled trials (RCTs) of surgical interventions represent the highest level of clinical evidence and often can lead to the widespread adoption of new surgical techniques. However, surgical RCTs are often undertaken in highly controlled clinical environments, in selected patients, and with procedures performed by a selected experienced group of surgeons, often in high-volume centers. Therefore, the generalizability of findings from surgical RCTs to the comparatively uncontrolled environment of national practice is questionable.^1,2^

The Laparoscopic versus Open Gastrectomy for Gastric Cancer trial published in 2021, recruited and randomized patients from 2015 to 2018 between laparoscopic and open gastrectomy.^3^ This multicenter RCT showed reduced blood loss in the laparoscopic group; however, operative time was longer. There were no significant differences between the groups in postoperative complications, in-hospital mortality, 30-day readmissions, R0 resection, median lymph node yield, 1-year overall survival, and global health-related quality of life. The primary outcome of this RCT was length of hospital stay, which laparoscopic gastrectomy failed to improve; however, importantly, the clinical and oncological safety of a laparoscopic approach was demonstrated within the trial.

For the patient, gastrectomy may be considered a technically less demanding operation with a lower complication rate when compared with esophagectomy.^4–6^ Furthermore, LOGICA included a robust surgical quality assurance program and the majority of Dutch centers performing gastrectomy, which may lead to broader and safer dissemination of laparoscopic gastrectomy within the Netherlands. Thus, the external validity of the LOGICA trial may be more likely to be replicated when examining this issue through the national Dutch Upper Gastrointestinal Cancer Audit (DUCA).^7^

The aim of this present study was to examine the influence of the LOGICA trial upon the practice and clinical outcomes from laparoscopic gastrectomy nationally within the Netherlands. To achieve this aim, specific objectives of this study were to (1) evaluate patient and tumor factors driving allocation to laparoscopic gastrectomy at a national level; (2) compare clinical outcomes from laparoscopic with open gastrectomy in both the RCT (LOGICA)^3^ and national clinical practice environments (DUCA)^7^; (3) examine changes over time in relation to the LOGICA trial (pre, during, and post), in the practice and outcomes from laparoscopic gastrectomy within the Netherlands.

## METHODS

### Data Sets

Data were obtained from the DUCA, which provided comprehensive data on all gastrectomies performed in the Netherlands between 2012 and 2021, and the LOGICA RCT, which ran from February 2015 to August 2018. The LOGICA data set included 46 variables, registering postoperative complications in accordance with the requirements of the DUCA registry at that time, and 516 variables were included within DUCA data set. All variables from the DUCA data set with >50% of data missing and those not relevant to the study objectives were excluded.

### Patients

All patients who underwent gastrectomy within the LOGICA RCT were included. From the DUCA data set, all patients who underwent a gastrectomy for gastric or gastroesophageal junction adenocarcinoma were included. We excluded all patients with missing data regarding overall complications (n=4), 30-day or in-hospital mortality (n=3), and surgical approach (n=1). The flowchart with inclusion and exclusion criteria is shown in Supplementary Fig. 1, Supplemental Digital Content 1, http://links.lww.com/SLA/E950. Patients who underwent a gastrectomy starting minimally invasive were classified as laparoscopic, as an intention-to-treat analysis was performed primarily, and a per-protocol analysis is presented within the Supplementary Material, Supplemental Digital Content 1, http://links.lww.com/SLA/E950.

### Statistical Analysis

Patient, tumor, and treatment characteristics of patients who underwent laparoscopic versus open gastrectomy were compared within the LOGICA and DUCA data sets using univariate analyses. These characteristics included sex, age, body mass index, American Society of Anesthesiologists score, Charlson Comorbidity Index, tumor location, cT, cN, cM, neoadjuvant treatment, surgical procedure, and year of surgery. Mann-Whitney *U* tests were used for continuous variables, and the *X* ^2^ or Fisher exact test was used for categorical variables.

Multilevel multivariable logistic regression analyses were performed to assess the effect of laparoscopic versus open gastrectomy upon clinical outcomes, including length of hospital stay, readmission, reoperation and endoscopic/radiologic reintervention, oncological outcomes, including resection radicality (R0) and number of resected lymph nodes; and complications including; intraoperative complications, overall complications, severe complications ≥Clavien-Dindo grade IIIa), anastomotic leakage, cardiac complications, pulmonary complications, pneumonia, and 30-day/in-hospital mortality), while adjusting for patient, tumor and treatment variables, as described above. To adjust for potential changes over time, year of surgery was added as a random effect factor within the multivariate model in the event the log-likelihood ratio test showed a better fit compared with the original model. In case of insufficient degrees of freedom to correct for the entire correction model, only confounders leading to a >10% change in odds ratio of the outcome were included in the multivariate model. These analyses were repeated to assess the effect of laparoscopic gastrectomy upon outcomes before (January 2012 to January 2015), during (February 2015 to August 2018), and after (September 2018 to December 2021) the LOGICA trial in the DUCA data set. Subgroup analyses were performed to match the DUCA data set to the inclusion and exclusion criteria of the LOGICA trial (curative resection, D2-lymphadenectomy, and M0), to classify conversions in the open cohort (per-protocol), and by excluding the first 20 laparoscopic gastrectomy cases of each center in the DUCA data set. Overall, *P* values were estimated using ANOVA analyses. All *P* values <0.05 were considered significant. Multicollinearity of variables was assessed by calculating the variance inflation factor, with a variance inflation factor of ≤2.5 considered indicative of the absence of multicollinearity. All outcomes were specifically registered through all years of the DUCA data set, except for pneumonia, which was only registered separately from 2016 onward. Patients with missing outcome data were excluded from the analysis. All statistical analyses were performed, and all figures were generated using R-studio version 4.2.1.^8^

## RESULTS

### Baseline Characteristics

**FIGURE 1:**
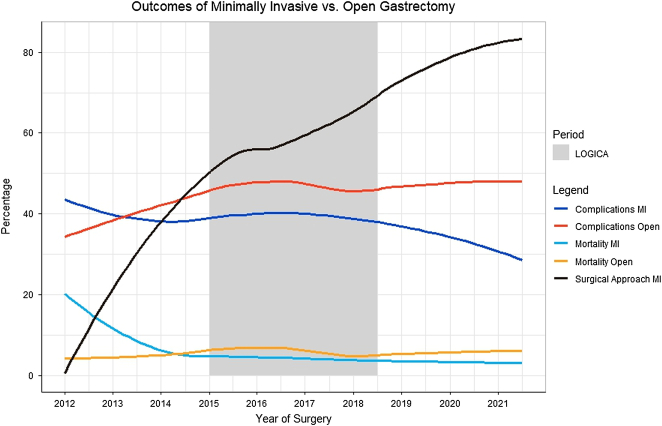
Overall complication and 30-day/in-hospital mortality rates after minimally invasive (MI) versus open gastrectomy, as well as the percentage of minimally invasive resections of all gastrectomies. The time frame of the LOGICA trial is represented by the gray background fill.

In total, 211 patients from the LOGICA RCT (105 open vs 106 laparoscopic) and 4131 patients from the DUCA data set (1884 open vs 2247 laparoscopic) were included. In the LOGICA RCT, patients who underwent laparoscopic gastrectomy had a significantly higher body mass index and more often did not receive neoadjuvant therapy when compared with open gastrectomy. At a national level, the DUCA database showed laparoscopic gastrectomy was used more often in female patients, higher American Society of Anesthesiologist-score patients, and M0 patients when compared with open surgery. Further significant differences were seen in age, Charlson Comorbidity Index, tumor location, cT, cN, neoadjuvant therapy, and year of surgery (Table 1). In 2012, laparoscopic gastrectomy was performed in 6% of patients before sharply rising, with a small decline in 2017, to 82% of patients in 2021 (Fig. 1).

**TABLE 1:** Patient, Tumor, and Treatment Characteristics of Patients That Underwent Laparoscopic Versus Open Gastrectomy in the LOGICA and DUCA Data Sets

	LOGICA			DUCA		
Characteristic	Open, N=105^*^, n (%)	Laparoscopic, N=106^*^, n (%)	*P* ^†^	Open, N=1884^*^, n (%)	Laparoscopic, N=2247^*^, n (%)	*P* ^†^
Sex	—	—	0.346	—	—	**<0.001**
Male	69 (66)	63 (59)	—	1231 (65)	1356 (60)	—
Female	36 (34)	43 (41)	—	653 (35)	891 (40)	—
Age	70 (60, 75)	70 (62, 76)	0.593	70 (62, 77)	72 (63, 78)	**0.005**
Age categories (y)	—	—	0.721	—	—	0.057
<65	38 (36)	33 (31)	—	597 (32)	643 (29)	—
65–75	41 (39)	46 (43)	—	696 (37)	835 (37)	—
>75	26 (25)	27 (25)	—	590 (31)	767 (34)	—
BMI	—	—	**0.002**	—	—	0.078
<20	11 (10)	2 (1.9)	—	145 (8)	165 (7)	—
20–25	54 (51)	58 (55)	—	965 (51)	1211 (54)	—
26–30	34 (32)	26 (25)	—	570 (30)	606 (27)	—
>30 kg	6 (5.7)	20 (19)	—	176 (9)	236 (11)	—
Missing	—	—	—	28 (2)	29 (1)	—
ASA-score	—	—	0.498	—	—	**<0.001**
1–2	77 (73)	82 (77)	—	1232 (65)	1383 (62)	—
3+	28 (27)	24 (23)	—	633 (34)	858 (38)	—
Missing	—	—	—	19 (1)	6 (<1)	—
Charlson comorbidity index	—	—	—	—	—	**0.002**
0	—	—	—	823 (44)	894 (40)	—
1	—	—	—	401 (21)	580 (26)	—
2+	—	—	—	660 (35)	773 (34)	—
Tumor location	—	—	0.856	—	—	**<0.001**
Fundus	10 (9.5)	12 (11)	—	129 (7)	173 (8)	—
Corpus	33 (31)	35 (33)	—	568 (30)	693 (31)	—
Antrum/pylorus	62 (59)	59 (56)	—	860 (46)	1107 (50)	—
Total stomach	—	—	—	112 (6)	83 (4)	—
Rest stomach/anastomosis	—	—	—	107 (6)	24 (1)	—
Unknown location	—	—	—	22 (1)	24 (1)	—
Gastro-esophageal junction	—	—	—	75 (4)	113 (5)	—
Missing	—	—	—	11	30	—
Clinical tumor stage	—	—	0.419	—	—	**<0.001**
T0–2	35 (33)	41 (39)	—	519 (28)	609 (27)	—
T3–4	70 (67)	65 (61)	—	944 (50)	1297 (58)	—
Tx	—	—	—	399 (21)	335 (15)	—
Missing	—	—	—	22 (1)	6 (<1)	—
Clinical node stage	—	—	0.372	—	—	**<0.001**
N0	54 (51)	61 (58)	—	939 (50)	1198 (53)	—
N+	51 (49)	45 (42)	—	740 (39)	895 (40)	—
Nx	—	—	—	183 (10)	147 (7)	—
Missing	—	—	—	22 (1)	7 (<1)	—
Clinical metastasis stage	—	—	0.999	—	—	**<0.001**
M0	105 (100)	106 (100)	—	1718 (91)	2114 (94)	—
M+	—	—	—	77 (4)	33 (2)	—
Mx	—	—	—	89 (5)	100 (5)	—
Neoadjuvant therapy	—	—	**0.021**	—	—	**<0.001**
Chemotherapy	85 (81)	71 (67)	—	1072 (57)	1313 (58)	—
None	20 (19)	35 (33)	—	766 (41)	827 (37)	—
Chemoradiotherapy	—	—	—	39 (2)	98 (4)	—
Radiotherapy	—	—	—	1 (<1)	1 (<1)	—
Missing	—	—	—	6 (<1)	8 (<1)	—
Surgical procedure	—	—	0.359	—	—	0.798
Total gastrectomy	41 (39)	48 (45)	—	856 (45)	1012 (45)	—
Partial gastrectomy	64 (61)	58 (55)	—	1028 (55)	1235 (55)	—
Year of surgery	—	—	0.987	—	—	**<0.001**
2012–2013	—	—	—	674 (36)	129 (6)	—
2014–2015	9 (9)	10 (9)	—	496 (26)	451 (20)	—
2016–2017	64 (61)	63 (59)	—	355 (19)	477 (21)	—
2018–2019	32 (30)	33 (31)	—	213 (11)	582 (26)	—
2020–2021	—	—	—	146 (8)	608 (27)	—

Bold values are statistically significant.

* n (%); Median (IQR).

† Pearson χ^2^ test; Wilcoxon rank sum test; Fisher exact test.

### Effects of Laparoscopic Gastrectomy on Clinical Outcomes Compared With Open Gastrectomy

Besides a significantly reduced risk of an in-hospital stay above the median of 7 days [adjusted odds ratio (aOR): 0.56; 95% CI: 0.31–0.99], no significant effect of laparoscopic gastrectomy on postoperative clinical outcomes was observed within the LOGICA RCT (Table 2). At a national level within the DUCA data set, laparoscopic gastrectomy was associated with a significant reduction in overall complications (aOR: 0.75; 95% CI: 0.65–0.87), severe complications (aOR: 0.84; 95% CI: 0.71–0.99), cardiac complications (aOR: 0.74; 95% CI: 0.55–0.99), and in-hospital stay above the median of 8 days (aOR: 0.41; 95% CI: 0.35–0.48), with a greater probability of a radical (R0) resection (aOR: 1.28; 95% CI: 1.03–1.59). However, laparoscopic gastrectomy was associated with an increase in endoscopic/radiologic reinterventions (aOR: 1.46; 95% CI: 1.02–2.13), with no effect upon reoperations. No significant effect of laparoscopic gastrectomy on all other outcomes (intraoperative complications, 30-day/in-hospital mortality, anastomotic leak, pulmonary complications, pneumonia, readmissions, and >15 resected lymph nodes) were observed over the entire study period in multivariate analyses.

**TABLE 2:** Multivariate Comparison of Clinical and Pathologic Outcomes of Laparoscopic Versus Open Gastrectomy in the LOGICA and DUCA Data Sets

	LOGICA				DUCA			
Characteristic	N	Open, N=105^*^, n (%)	Laparoscopic, N=106^*^, n (%)	aOR^†^	N	Open, N=1884^*^, n (%)	Laparoscopic, N=2247^*^, n (%)	aOR^†^
Intraoperative complications	211	8 (8)	6 (6)	0.73 (0.23–2.17)	4118	67 (4)	81 (4)	1.20 (0.83–1.75)
30-day/in-hospital mortality	211	7 (7)	5 (5)	0.69 (0.20–2.24)	4131	96 (5)	95 (4)	0.86 (0.64–1.16)
All overall complications	211	44 (42)	48 (45)	1.03 (0.59–1.80)^‡^	4131	802 (43)	826 (37)	**0.75 (0.65–0.87)**
Severe complications (≥CD3)	211	23 (22)	19 (18)	0.67 (0.33–1.34)^‡^	4131	393 (21)	414 (18)	**0.84 (0.71–0.99)**
Anastomotic leak	211	10 (10)	10 (9.4)	0.73 (0.26–1.97)^‡^,^§^	4131	143 (8)	175 (8)	1.02 (0.79–1.32)
Cardiac complications	211	7 (7)	12 (11)	1.60 (0.60–4.53)^‡^	4130	116 (6)	114 (5)	0.74 (0.55–0.99)
Pulmonary complications	211	15 (14)	17 (16)	0.96 (0.44–2.12)^∥^	4130	302 (16)	313 (14)	0.83 (0.69–1.00)
Pneumonia	211	13 (12)	16 (15)	1.03 (0.45–2.35)^∥^	1751	91 (13)	171 (10)	0.78 (0.58-1.04)
Reoperations	211	15 (14)	13 (12)	0.74 (0.32–1.68)^‡^	4131	238 (13)	261 (12)	0.90 (0.74–1.10)
Endoscopic/radiologic reintervention	211	—	—	—	4131	50 (3)	86 (4)	**1.46 (1.02–2.13)**
Length of hospital stay (over median of 8 days)	211	51 (49)	40 (38)	**0.56 (0.31–0.99)** ^§^	4121	1099 (58)	1751 (33)	**0.41 (0.35–0.48)**
Readmissions	211	10 (10)	11 (10)	1.22 (0.48–3.08)^‡^	4077	224 (12)	294 (13)	1.12 (0.92–1.36)
Resection radicality	211	100 (95)	101 (95)	0.99 (0.27–3.66)	4131	1625 (86)	1994 (89)	**1.28 (1.03–1.59)**
Resected lymph nodes (median)	211	29 (22,39)	29 (21,37)	*P*=0.486^¶^	4128	21 (14,29)	23 (17,32)	***P*** **<0.001** ^¶^
>15 resected lymph nodes	211	100 (95)	100 (94)	1.01 (0.28–3.61)^∥^	4128	1404 (75)	1928 (86)	1.06 (0.86–1.29)

Bold values are statistically significant.

* n (%); median (IQR).

† Adjusted odds ratio. Corrected for: sex, age, BMI, Charlson Comorbidity Index, ASA-score, tumor location, cT stage, cN stage, cM stage, neoadjuvant therapy, type of gastrectomy, and year of surgery as random effect factor. In case of insufficient degrees of freedom for correction for the entire correction model, only confounders leading to a 10% change in odds ratio were included in the multivariable model. Year of surgery was added as random effect factor to the model in case the log-likelihood ratio test showed a better fit compared with the original multivariable model.

‡ Neoadjuvant therapy.

§ Surgical procedure.

∥ BMI.

¶ Wilcoxon rank sum test.

BMI indicates body mass index.

Subgroup analyses of matching the DUCA data set to the inclusion and exclusion criteria of the LOGICA RCT showed consistent results with clinical benefits observed with laparoscopic gastrectomy, including significantly less overall and cardiac complications and a lower risk of an in-hospital stay above the median of 8 days (Supplementary Table 1, Supplemental Digital Content 1, http://links.lww.com/SLA/E950). In addition, a significant reduction in 30-day/in-hospital mortality was observed in this cohort. Further subgroup per-protocol analyses of converted laparoscopic resections classified as open surgery showed a significant reduction in intraoperative, overall, severe, and cardiac complications, pneumonia and length of hospital stay, as well as a significant increase in resection radicality and >15 lymph nodes harvested associated with laparoscopic gastrectomy (Supplementary Table 2, Supplemental Digital Content 1, http://links.lww.com/SLA/E950). Subgroup analyses of excluding the first 20 laparoscopic gastrectomy from each center to match the center inclusion criteria for the LOGICA trial showed similar overall findings with significant improvements in overall, severe, and cardiac complications; length of hospital stay and resection radicality; and lymph node harvest. However, the rate of endoscopic and radiologic reintervention was increased in the laparoscopic gastrectomy group (Supplementary Table 3, Supplemental Digital Content 1, http://links.lww.com/SLA/E950).

### Effects of Laparoscopic Gastrectomy on Outcomes Before, During, and After the LOGICA Trial

**FIGURE 2:**
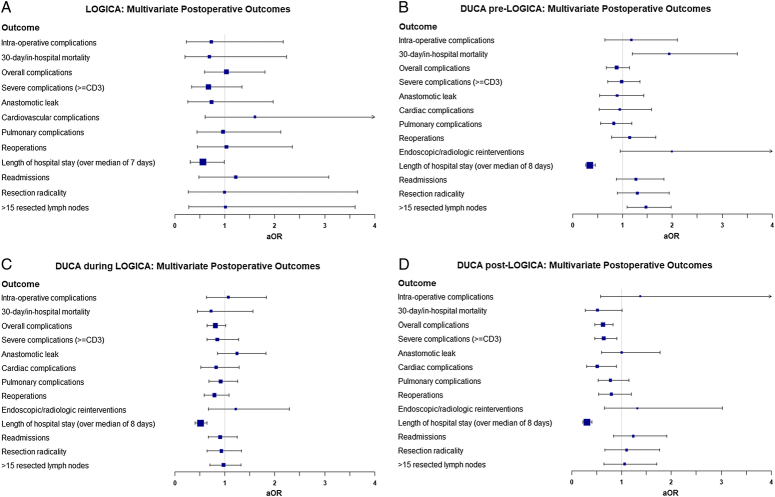
Forest plots showing the effect of laparoscopic versus open gastrectomy on postoperative clinical outcomes during: the LOGICA RCT (A); the DUCA data set before the start of the LOGICA RCT (B); the DUCA data set during the LOGICA RCT (C); the DUCA data set after the end of the LOGICA RCT (D).

Figure 1 shows the overall complication and 30-day/in-hospital mortality rate of laparoscopic and open gastrectomy over time. Both the overall complication and mortality rates of laparoscopic gastrectomy started above those of open gastrectomy before descending below it in 2013 and 2014, respectively. The overall complication rate from laparoscopic gastrectomy accelerated its decline after the end of the LOGICA trial. Before the start of LOGICA, laparoscopic gastrectomy was associated with an increased 30-day/in-hospital mortality (aOR: 1.94; 95% CI: 1.20–3.30) at a national level (Fig. 2; Supplementary Table 4, Supplemental Digital Content 1, http://links.lww.com/SLA/E950). This effect was not observed during and after the LOGICA trial (Fig. 2; Supplementary Tables 5, Supplemental Digital Content 1, http://links.lww.com/SLA/E950 and S6, Supplemental Digital Content 1, http://links.lww.com/SLA/E950). No further effect of laparoscopic gastrectomy on complications was observed before the LOGICA trial. However, a gradual shift towards a beneficial effect of laparoscopic gastrectomy upon complications was observed, reaching a significant reduction in overall complications (aOR: 0.62; 95% CI: 0.46–0.82), severe complications (aOR: 0.64; 95% CI: 0.46–0.90) and cardiac complications (aOR: 0.51; 95% CI: 0.30–0.89) after the LOGICA trial. The benefit of laparoscopic gastrectomy upon hospital stay was prevalent through all periods.

## DISCUSSION

The results of the LOGICA randomized controlled trial across 10 experienced upper gastrointestinal Dutch centers showed that laparoscopic gastrectomy was associated with no significant improvements in length of hospital stay or postoperative complications when compared with open gastrectomy.^3^ Analysis of the DUCA from 2012 to 2021 showed that laparoscopic gastrectomy was associated with reductions in overall and severe complications, and specifically cardiac and pulmonary complications, as well as a reduction in the proportion of patients with a length of hospital stay of longer than 8 days. Importantly, during the study period, there was a substantial rise in the proportion of patients undergoing laparoscopic gastrectomy within the Netherlands from 6% in 2012 to 82% in 2021 (Fig. 1). Interestingly, short-term outcomes from laparoscopic gastrectomy when compared with open gastrectomy showed a continuous pattern of improvement largely from being equivalent before and during the LOGICA trial to superior after the LOGICA trial (Fig. 2). Oncological outcomes (resection margin and lymph node harvest) were similar between the 2 techniques across all analyses both within the RCT (LOGICA) and in national practice (DUCA). The results of this study examining the external validity of the LOGICA RCT within the DUCA data set suggest that the RCT may act as vehicle to facilitate the safe dissemination of a challenging surgical technique such as laparoscopic gastrectomy. With the potential improvements in clinical outcomes only observed after the trial, once surgeons are well beyond their learning curve with complex surgical interventions.

When considering the external validity of a RCT concerning surgical interventions in surgical oncology, it is essential to remain acutely aware of the complexity of the intervention under investigation and the mechanism for its implementation within and following the RCT. Our research group previously examined the external validity of the TIME trial, which showed a substantial reduction in pulmonary complications associated with totally minimally invasive compared with open esophagectomy.^1^ When examining the DUCA data set after the TIME trial, minimally invasive esophagectomy was associated with an increase in pulmonary complications and reoperations.^2^ The results of this study reflected nonexpert surgeons adopting this technique in a nonstandardized fashion, going through their learning curve in an unregulated manner, resulting in substantial patient complications.

There are important differences between this present study concerning the LOGICA trial and the previously conducted study concerning the TIME trial.^3^ The TIME trial was conducted across 5 European centers in 3 European countries and involved the more complex surgical procedure of totally minimally invasive esophagectomy, while the LOGICA trial was conducted across 10 Dutch centers and involved a less complex procedure of laparoscopic gastrectomy. The LOGICA trial benefited from a robustly developed surgical quality assurance (SQA) program, which included, before inclusion in the trial, standardization of the surgical technique with all surgeons having completed the European Society of Surgical Oncology laparoscopic gastrectomy training program.^9^ Furthermore, each surgical team had to have performed at least 20 laparoscopic gastrectomies,^10^ and 2 procedural videos were reviewed and approved by the trial principal investigators, before entry into the trial. Surgical performance and specifically completeness of lymphadenectomy were monitored during the trial through intraoperative photos. The broad inclusion of centers within the LOGICA trial (10 out 15 performing gastrectomy within the Netherlands), along with the robust SQA program, facilitated the safe national dissemination of laparoscopic gastrectomy when compared with the results seen after the TIME trial, as described above.^2^

The learning or proficiency gain curve is well described in the surgical literature and describes adverse patient outcomes as surgeon gain proficiency in performing a surgical technique.^11,12^ In upper gastrointestinal cancer surgery, along with other research groups, we have shown the adverse effects upon short-term and long-term mortality that can be seen at a national level as surgeons go through their learning curve.^12–15^ Furthermore, the importance of SQA with randomized controlled trials is well established in standardizing surgical performance within the trial and attempting to mitigate the surgical learning curve effects.^16–18^ With an increase in the development of video and photo analytical tools, along with a wider availability of intraoperative video recording and proctoring program, SQA protocols are increasingly becoming established as the standard of practice in surgical RCTs.^19–21^ Given the results from laparoscopic gastrectomy observed within the LOGICA trial and within the DUCA data set after completion of the trial, it could perhaps be explained that surgeons were still within their learning curve during the LOGICA trial, and their performance and outcomes improved upon completion of the trial. These findings speak more broadly to the importance of a clear strategy for the dissemination of a surgical technique beyond a RCT to clinical practice upon completion of the RCT.^22^ The LOGICA trial serves as a good example of clear planning of a dissemination strategy with a robust SQA program embedded within the trial. Other examples include the UK national LAPCO training program, which facilitated the safe adoption of laparoscopic colorectal surgery across the United Kingdom.^23,24^ Further, the absence of clear, safe dissemination strategies, including SQA for complex techniques in surgical oncology can lead substantial patient harm, and in some instances, abandonment of new techniques as was seen within transanal total mesorectal excision in the absence of randomized data, showing a benefit to the technique.^25^

Given the importance of the findings of this present study it is crucial to consider the strengths and limitations in terms of study design. First, this study is novel in that it has shown the importance of a well-conducted RCT with a robust SQA program in facilitating the dissemination of a challenging surgical procedure nationally. Second, the data analyzed were collated from 2 well-validated sources, a well-designed RCT and a large well-validated national data set concerning surgery for gastric cancer. Third, given the underlying differences in patient and tumor characteristics between the study groups, the analysis conducted was appropriate with adjustment for known and measured confounding factors. The main limitations of the study include the potential effect of unmeasured confounding factors that evolved during the study period, including the introduction of enhanced recovery protocols and the ongoing centralization of cancer surgical services to high-volume centers within the Netherlands. Specific improvements in perioperative care and intraoperative and postoperative technology may also have contributed to improvements in clinical outcomes over the study period observed in the laparoscopic gastrectomy group. However, given the lack of improvement in postoperative morbidity seen from open gastrectomy over the study period (Fig. 1), these changes are unlikely to have influenced the results seen. A further limitation is that within the period from February 2015 to August 2018 of the LOGICA trial, we were unable to separately identify and analyze those patients within the trial from those operated on outside of the trial within the same period of time, which would be an interest area for further research. Furthermore, drivers for allocation to laparoscopic gastrectomy will have varied across centers over the study period and this reflects assessment of “real-world” national practice. However, the dramatic rise seen in laparoscopic gastrectomy over the study following the LOGICA trial was impressive and does suggest a fundamental adoption of the technique nationally may have been facilitated by the trial.

In conclusion, the result of this study suggests the wider benefits of the LOGICA trial include safe dissemination of laparoscopic gastrectomy across the Netherlands, and further ongoing progression through the surgical learning curve may lead to the true benefit of a technique being observed only after the evaluating trial. The importance of a robust SQA program in the design of the RCT is crucial to facilitate the national dissemination of the technique following the trial and reducing potential patient harm during the surgeons learning curve.

## Supplementary Material
