# Supplementary material for: Evolution in Laparoscopic Gastrectomy From a Randomized Controlled Trial Through National Clinical Practice
Source: Ann Surg. 2023 Nov 23;279(3):394–401. doi: 10.1097/SLA.0000000000006162 (PMC10829898; doi:10.1097/SLA.0000000000006162)
Supplement: Supplementary file 1 [file sla-279-394-s001.docx]

**Supplementary material**


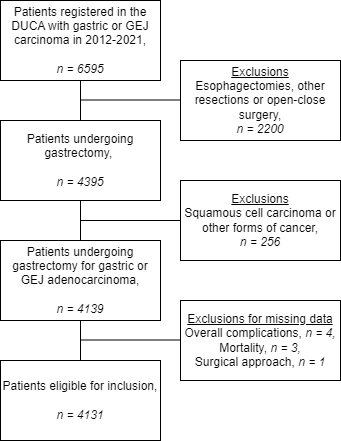


**Supplementary Figure 1**: Flowchart of in- and exclusions

| **Supplementary Table 1:** Multivariate comparison of clinical and pathological outcomes of laparoscopic versus open gastrectomy in the DUCA dataset matched to the in- and exclusion criteria of the LOGICA trial. | | | | | | |
| --- | --- | --- | --- | --- | --- | --- |
| **Characteristic** | **N** | **Overall**,  N = 1,877^1^ | **Open**,  N = 522^1^ | **Laparoscopic**,  N = 1,355^1^ | **aOR^2^** | **p-value** |
| **Intra-operative complications** | 1,877 | 58 (3.1%) | 16 (3.1%) | 42 (3.1%) | 0.98 (0.55-1.81) | 0.937 |
| **30-day/in-hospital mortality** | 1,877 | 77 (4.1%) | 31 (5.9%) | 46 (3.4%) | 0.56 (0.36-0.91) | 0.019 |
| **All overall complications** | 1,877 | 733 (39%) | 231 (44%) | 502 (37%) | 0.78 (0.62-0.97) | 0.024 |
| **Severe complications (>=CD3)** | 1,877 | 367 (20%) | 111 (21%) | 256 (19%) | 0.88 (0.68-1.16) | 0.361 |
| **Anastomotic leak^a^** | 1,877 | 151 (8.0%) | 39 (7.5%) | 112 (8.3%) | 1.26 (0.86-1.89) | 0.241 |
| **Cardiac complications** | 1,877 | 99 (5.3%) | 37 (7.1%) | 62 (4.6%) | 0.62 (0.41-0.95) | 0.028 |
| **Pulmonary complications** | 1,877 | 272 (14%) | 89 (17%) | 183 (14%) | 0.80 (0.59-1.08) | 0.145 |
| **Reoperations** | 1,877 | 228 (12%) | 74 (14%) | 154 (11%) | 0.78 (0.57-1.08) | 0.132 |
| **Endoscopic/radiologic reintervention^b^** | 1,877 | 71 (3.8%) | 18 (3.4%) | 53 (3.9%) | 1.39 (0.79-2.58) | 0.256 |
| **Length of hospital stay (over median of 8 days)** | 1,877 | 669 (36%) | 272 (52%) | 397 (29%) | 0.42 (0.33-0.52) | <0.001 |
| **Readmissions** | 1,861 | 260 (14%) | 69 (13%) | 191 (14%) | 1.03 (0.76-1.41) | 0.839 |
| **Resection radicality** | 1,877 | 1,694 (90%) | 477 (91%) | 1,217 (90%) | 0.96 (0.64-1.40) | 0.823 |
| **>15 resected lymph nodes** | 1,877 | 1,703 (91%) | 469 (90%) | 1,234 (91%) | 0.77 (0.51-1.15) | 0.202 |
| ^1^ n (%); Median (IQR)  ^2^ Adjusted Odds Ratio. Corrected for: sex, age, BMI, Charlson Comorbidity Index, ASA-score, tumor location, cT stage, cN stage, cM stage, neo-adjuvant therapy, type of gastrectomy, and year of surgery as random effect factor. In case of insufficient degrees of freedom for correction for the entire correction model, only confounders leading to a 10% change in odds ratio were included in the multivariate model. Year of surgery was added as random effect factor to the model in case the log-likelihood ratio test showed a better fit compared to the original multivariate model.  Adjusted for: ^a^Surgical procedure; ^b^Tumor location | | | | | | |

| **Supplementary Table 2**: Multivariate comparison of clinical and pathological outcomes of laparoscopic versus open gastrectomy in the DUCA dataset with conversions classified as open surgery (per-protocol). | | | | | | |
| --- | --- | --- | --- | --- | --- | --- |
| **Characteristic** | **N** | **Overall**,  N = 4,131^1^ | **Open**,  N = 2,102^1^ | **Laparoscopic**,  N = 2,029^1^ | **aOR^2^** | **p-value** |
| **Intra-operative complications** | 4,118 | 148 (3.6%) | 98 (4.7%) | 50 (2.5%) | 0.68 (0.51-0.90) | <0.001 |
| **30-day/in-hospital mortality** | 4,131 | 191 (4.6%) | 111 (5.3%) | 80 (3.9%) | 0.78 (0.57-1.05) | 0.101 |
| **All overall complications** | 4,131 | 1,628 (39%) | 903 (43%) | 725 (36%) | 0.72 (0.63-0.83) | <0.001 |
| **Severe complications (>=CD3)** | 4,131 | 807 (20%) | 451 (21%) | 356 (18%) | 0.77 (0.65-0.91) | 0.002 |
| **Anastomotic leak** | 4,131 | 318 (7.7%) | 163 (7.8%) | 155 (7.6%) | 1.01 (0.78-1.30) | 0.956 |
| **Cardiac complications** | 4,130 | 230 (5.6%) | 136 (6.5%) | 94 (4.6%) | 0.65 (0.48-0.86) | 0.003 |
| **Pulmonary complications** | 4,130 | 615 (15%) | 342 (16%) | 273 (13%) | 0.81 (0.67-0.97) | 0.021 |
| **Reoperations** | 4,131 | 499 (12%) | 269 (13%) | 230 (11%) | 0.87 (0.71-1.07) | 0.188 |
| **Endoscopic/radiologic reintervention** | 4,131 | 136 (3.3%) | 58 (2.8%) | 78 (3.8%) | 1.39 (0.97-1.99) | 0.073 |
| **Length of hospital stay (over median of 8 days)** | 4,121 | 1,850 (45%) | 1,212 (58%) | 638 (32%) | 0.39 (0.33-0.45) | <0.001 |
| **Readmissions** | 4,077 | 518 (13%) | 258 (12%) | 260 (13%) | 1.07 (0.88-1.29) | 0.518 |
| **Resection radicality** | 4,131 | 3,619 (88%) | 1,803 (86%) | 1,816 (90%) | 1.54 (1.24-1.93) | <0.001 |
| **>15 resected lymph nodes** | 4,128 | 3,332 (81%) | 1,570 (75%) | 1,762 (87%) | 1.24 (1.01-1.51) | 0.004 |
| ^1^ n (%); Median (IQR)  ^2^ Adjusted Odds Ratio. Corrected for: sex, age, BMI, Charlson Comorbidity Index, ASA-score, tumor location, histology, clinical Tumor stage, clinical Node stage, neoadjuvant therapy and type of gastrectomy and year of surgery as random effect factor. In case of insufficient degrees of freedom for correction for all possible confounders, only confounders leading to a 10% change in OR were included for analyses. Year of surgery was added as random effect to the model in case the log-likelihood ratio test showed a better fit compared to the original multivariable model. | | | | | | |

***Supplementary* Table 3:** Multivariate comparison of clinical and pathological outcomes of laparoscopic versus open gastrectomy in the DUCA dataset excluding the first 20 laparoscopic gastrectomy cases from each center and thus matched to the center inclusion for the LOGICA trial.

| **Characteristic** | **N** | **Overall**,  N = 3,682^1^ | **Open**,  N = 1,884^1^ | **Laparoscopic**,  N = 1,798^1^ | **aOR^2^** | **P-value^3^** |
| --- | --- | --- | --- | --- | --- | --- |
| **Intra-operative complications** | 3,671 | 126 (3%) | 67 (4%) | 59 (3%) | 0.88 (0.61-1.26) | 0.480 |
| **30-day/in-hospital mortality** | 3,682 | 164 (5%) | 96 (5%) | 68 (3%) | 0.79 (0.57-1.08) | 0.142 |
| **All overall complications** | 3,682 | 1,458 (40%) | 802 (43%) | 656 (36%) | **0.72 (0.62-0.85)** | **<0.001** |
| **Severe complications (>=CD3)** | 3,682 | 725 (20%) | 393 (21%) | 332 (18%) | **0.82 (0.68-0.97)** | **0.024** |
| **Anastomotic leak** | 3,682 | 277 (8%) | 143 (8%) | 134 (8%) | 0.97 (0.74-1.27) | 0.805 |
| **Cardiac complications** | 3,681 | 208 (6%) | 116 (6%) | 92 (5%) | **0.74 (0.54-0.99)** | **0.047** |
| **Pulmonary complications** | 3,681 | 557 (15%) | 302 (16%) | 255 (14%) | 0.84 (0.69-1.02) | 0.071 |
| **Reoperations** | 3,682 | 441 (12%) | 238 (13%) | 203 (11%) | 0.87 (0.70-1.08) | 0.195 |
| **Endoscopic/radiologic reintervention** | 3,682 | 124 (3%) | 50 (3%) | 74 (4%) | **1.61 (1.11-2.36)** | **0.012** |
| **Length of hospital stay (over median of 8 days)** | 3,677 | 1,648 (45%) | 1,099 (58%) | 549 (31%) | **0.39 (0.33-0.46)** | **<0.001** |
| **Readmissions** | 3,637 | 457 (13%) | 224 (12%) | 233 (13%) | 1.08 (0.88-1.33) | 0.457 |
| **Resection radicality** | 3,682 | 3,233 (88%) | 1,625 (86%) | 1,608 (89%) | **1.38 (1.09-1.74)** | **0.007** |
| **Resected lymph nodes (median)** | 3,680 | 23 (16, 31) | 21 (14, 29) | 24 (18, 33) | **-** | **[<0.001]^4^** |
| **>15 resected lymph nodes** | 3,680 | 2,984 (81%) | 1,404 (75%) | 1,580 (88%) | 1.16 (0.91-1.47) | 0.240 |
| \| ^1^ n (%); Median (IQR) \| \| --- \| \| ^2^ Adjusted Odds Ratio. Corrected for: sex, age, BMI, Charlson Comorbidity Index, ASA-score, tumor location, histology, clinical Tumor stage, clinical Node stage, neoadjuvant therapy and type of gastrectomy and year of surgery as random effect factor. In case of insufficient degrees of freedom for correction for all possible confounders, only confounders leading to a 10% change in OR were included for analyses. Year of surgery was added as random effect to the model in case the log-likelihood ratio test showed a better fit compared to the original multivariable model.  ^3^ Multilevel multivariable logistic regression  ^4^ Wilcoxon rank sum test \| | | | | | | |

| **Supplementary Table 4**: Multivariate comparison of clinical and pathological outcomes of laparoscopic versus open gastrectomy in the DUCA dataset before (January 2012 – January 2015) the LOGICA trial. | | | | | | |
| --- | --- | --- | --- | --- | --- | --- |
| **Characteristic** | **N** | **Overall**,  N = 1,413^1^ | **Open**,  N = 1,014^1^ | **Laparoscopic**,  N = 399^1^ | **aOR^2^** | **p-value** |
| **Intra-operative complications^a^** | 1,404 | 56 (4.0%) | 38 (3.8%) | 18 (4.5%) | 1.18 (0.64-2.10) | 0.580 |
| **30-day/in-hospital mortality^b^** | 1,413 | 71 (5.0%) | 46 (4.5%) | 25 (6.3%) | 1.94 (1.20-3.30) | 0.012 |
| **All overall complications** | 1,413 | 541 (38%) | 397 (39%) | 144 (36%) | 0.88 (0.67-1.14) | 0.331 |
| **Severe complications (>=CD3)** | 1,413 | 270 (19%) | 195 (19%) | 75 (19%) | 0.98 (0.70-1.35) | 0.886 |
| **Anastomotic leak^a^** | 1,413 | 104 (7.4%) | 72 (7.1%) | 32 (8.0%) | 0.89 (0.54-1.43) | 0.633 |
| **Cardiac complications** | 1,413 | 81 (5.7%) | 58 (5.7%) | 23 (5.8%) | 0.94 (0.53-1.58) | 0.812 |
| **Pulmonary complications** | 1,413 | 204 (14%) | 153 (15%) | 51 (13%) | 0.82 (0.56-1.19) | 0.306 |
| **Reoperations** | 1,413 | 168 (12%) | 116 (11%) | 52 (13%) | 1.15 (0.78-1.67) | 0.471 |
| **Endoscopic/radiologic reintervention^a^** | 1,413 | 38 (2.7%) | 20 (2.0%) | 18 (4.5%) | 1.99 (0.95-4.10) | 0.069 |
| **Length of hospital stay (under median of 8 days)** | 1,404 | 811 (58%) | 630 (62%) | 181 (46%) | 0.34 (0.26-0.45) | <0.001 |
| **Readmissions** | 1,398 | 160 (11%) | 109 (11%) | 51 (13%) | 1.27 (0.87-1.83) | 0.207 |
| **Resection radicality** | 1,413 | 1,203 (85%) | 853 (84%) | 350 (88%) | 1.30 (0.89-1.94) | 0.170 |
| **>15 resected lymph nodes** | 1,410 | 974 (69%) | 669 (66%) | 305 (77%) | 1.47 (1.09-1.98) | 0.011 |
| ^1^ n (%); Median (IQR)  ^2^ Adjusted Odds Ratio. Corrected for: sex, age, BMI, Charlson Comorbidity Index, ASA-score, tumor location, histology, clinical Tumor stage, clinical Node stage, neoadjuvant therapy and type of gastrectomy and year of surgery as random effect factor. In case of insufficient degrees of freedom for correction for all possible confounders, only confounders leading to a 10% change in OR were included for analyses. Year of surgery was added as random effect to the model in case the log-likelihood ratio test showed a better fit compared to the original multivariable model.  Adjusted for: ^a^Surgical procedure; ^b^Neo-adjuvant treatment | | | | | | |

| **Supplementary Table 5**: Multivariate comparison of clinical and pathological outcomes of laparoscopic versus open gastrectomy in the DUCA dataset during (February 2015 – August 2018) the LOGICA trial. | | | | | | |
| --- | --- | --- | --- | --- | --- | --- |
| **Characteristic** | **N** | **Overall**,  N = 1,409^1^ | **Open**,  N = 593^1^ | **Laparoscopic**,  N = 816^1^ | **aOR^2^** | **p-value** |
| **Intra-operative complications** | 1,405 | 59 (4.2%) | 24 (4.1%) | 35 (4.3%) | 1.07 (0.63-1.83) | 0.815 |
| **30-day/in-hospital mortality** | 1,409 | 74 (5.3%) | 36 (6.1%) | 38 (4.7%) | 0.72 (0.45-1.56) | 0.171 |
| **All overall complications** | 1,409 | 606 (43%) | 278 (47%) | 328 (40%) | 0.81 (0.64-1.02) | 0.074 |
| **Severe complications (>=CD3)** | 1,409 | 282 (20%) | 128 (22%) | 154 (19%) | 0.85 (0.64-1.28) | 0.257 |
| **Anastomotic leak^a^** | 1,409 | 129 (9.2%) | 51 (8.6%) | 78 (9.6%) | 1.24 (0.85-1.82) | 0.269 |
| **Cardiac complications** | 1,408 | 79 (5.6%) | 37 (6.2%) | 42 (5.1%) | 0.82 (0.52-1.29) | 0.388 |
| **Pulmonary complications** | 1,408 | 230 (16%) | 103 (17%) | 127 (16%) | 0.92 (0.68-1.26) | 0.598 |
| **Reoperations** | 1,409 | 184 (13%) | 85 (14%) | 99 (12%) | 0.79 (0.58-1.08) | 0.145 |
| **Endoscopic/radiologic reintervention^a,b^** | 1,409 | 50 (3.5%) | 20 (3.4%) | 30 (3.7%) | 1.22 (0.67-2.29) | 0.516 |
| **Length of hospital stay (over median of 8 days)** | 1,408 | 646 (46%) | 330 (56%) | 316 (39%) | 0.51 (0.40-0.64) | <0.001 |
| **Readmissions** | 1,380 | 187 (14%) | 83 (14%) | 104 (13%) | 0.91 (0.67-1.25) | 0.562 |
| **Resection radicality^b^** | 1,409 | 1,228 (87%) | 520 (88%) | 708 (87%) | 0.93 (0.64-1.33) | 0.679 |
| **>15 resected lymph nodes** | 1,409 | 1,176 (83%) | 490 (83%) | 686 (84%) | 0.97 (0.70-1.32) | 0.831 |
| ^1^ n (%); Median (IQR)  ^2^ Adjusted Odds Ratio. Corrected for: sex, age, BMI, Charlson Comorbidity Index, ASA-score, tumor location, histology, clinical Tumor stage, clinical Node stage, neoadjuvant therapy and type of gastrectomy and year of surgery as random effect factor. In case of insufficient degrees of freedom for correction for all possible confounders, only confounders leading to a 10% change in OR were included for analyses. Year of surgery was added as random effect to the model in case the log-likelihood ratio test showed a better fit compared to the original multivariable model.  Adjusted for: ^a^Surgical procedure; ^b^Tumor location | | | | | | |

| **Supplementary Table 6:** Multivariate comparison of clinical and pathological outcomes of laparoscopic versus open gastrectomy in the DUCA dataset after (September 2018– December 2021) the LOGICA trial. | | | | | | |
| --- | --- | --- | --- | --- | --- | --- |
| **Characteristic** | **N** | **Overall**,  N = 1,306^1^ | **Open**,  N = 277^1^ | **Laparoscopic**,  N = 1,029^1^ | **aOR^2^** | **p-value** |
| **Intra-operative complications** | 1,306 | 33 (2.5%) | 5 (1.8%) | 28 (2.7%) | 1.38 (0.57-4.13) | 0.498 |
| **30-day/in-hospital mortality^c^** | 1,306 | 46 (3.5%) | 14 (5.1%) | 32 (3.1%) | 0.51 (0.27-1.01) | 0.054 |
| **All overall complications** | 1,306 | 481 (37%) | 127 (46%) | 354 (34%) | 0.62 (0.46-0.82) | 0.001 |
| **Severe complications (>=CD3)** | 1,306 | 255 (20%) | 70 (25%) | 185 (18%) | 0.64 (0.46-0.90) | 0.011 |
| **Anastomotic leak^a,b^** | 1,306 | 85 (6.5%) | 20 (7.2%) | 65 (6.3%) | 1.00 (0.59-1.78) | 0.999 |
| **Cardiac complications^c^** | 1,306 | 70 (5.4%) | 21 (7.6%) | 49 (4.8%) | 0.51 (0.30-0.89) | 0.019 |
| **Pulmonary complications** | 1,306 | 181 (14%) | 46 (17%) | 135 (13%) | 0.77 (0.53-1.15) | 0.201 |
| **Reoperations** | 1,306 | 147 (11%) | 37 (13%) | 110 (11%) | 0.79 (0.54-1.20) | 0.271 |
| **Endoscopic/radiologic reintervention^b^** | 1,306 | 48 (3.7%) | 10 (3.6%) | 38 (3.7%) | 1.32 (0.65-3.02) | 0.459 |
| **Length of hospital stay (over median of 8 days)** | 1,306 | 392 (30%) | 139 (50%) | 253 (25%) | 0.30 (0.22-0.40) | <0.001 |
| **Readmissions** | 1,296 | 171 (13%) | 32 (12%) | 139 (14%) | 1.24 (0.83-1.91) | 0.293 |
| **Resection radicality** | 1,306 | 1,185 (91%) | 252 (91%) | 933 (91%) | 1.10 (0.66-1.77) | 0.695 |
| **>15 resected lymph nodes** | 1,306 | 1,179 (90%) | 245 (88%) | 934 (91%) | 1.06 (0.64-1.71) | 0.813 |
| ^1^ n (%); Median (IQR)  ^2^ Adjusted Odds Ratio. Corrected for: sex, age, BMI, Charlson Comorbidity Index, ASA-score, tumor location, histology, clinical Tumor stage, clinical Node stage, neoadjuvant therapy and type of gastrectomy and year of surgery as random effect factor. In case of insufficient degrees of freedom for correction for all possible confounders, only confounders leading to a 10% change in OR were included for analyses. Year of surgery was added as random effect to the model in case the log-likelihood ratio test showed a better fit compared to the original multivariable model.  Adjusted for: ^a^Surgical procedure; ^b^Tumor location; ^c^Age category | | | | | | |
